# Nomophobia, phubbing and depression: A cohort comparison (young adults and middle-aged adults)

**DOI:** 10.1192/j.eurpsy.2026.10860

**Published:** 2026-07-31

**Authors:** I. Faria, B. R. Maia

**Affiliations:** 1 Faculty of Philosophy and Social Sciences; 2 Faculty of Philosophy and Social Sciences, Centre for Philosophical and Humanistic Studies, Universidade Católica Portuguesa, Braga, Portugal

## Abstract

**Introduction:**

Attachment to mobile phones may trigger depressive symptoms and depression episodes.

**Objectives:**

This study aims to explore the relationship between nomophobia, phubbing and depression in young adults and in middle-aged adults.

**Methods:**

Two-hundred and twenty Portuguese subjects (young adults: *n* = 186, 84.5%; middle-aged adults: *n* = 34, 15.5%), with a mean age of 27 years old (*SD* = 9.94; range = 18-59 years) completed the Nomophobia Questionnaire, The Phubbing Scale, The Depression, Anxiety, and Stress Scale and the Clinical Depression Assessment Inventory.

**Results:**

The cohort of young adults shows higher levels of nomophobia (severe: 16.1%, *n* = 30; moderate: 41.9%, *n* = 78), phubbing, depressive symptoms and depression. In the cohort of middle-aged adults, the majority showed mild nomophobia (64.7%, n = 22). In both cohorts, the absence of homophobia was of 0%. In young-adults and in middle-aged cohorts, nomophobia and phubbing were significantly correlated with depressive symptoms (*r*= .30** .38**; and *r*=.31; .39**, respectively) and with clinical depression (*r*=.34**, *r* = .34**; and *r*= .49**, *r*=.57**, respectively). Young-adult cohort (*Md* = 4, *n* = 186) presented significantly higher scores of depressive symptoms than middle aged cohort (*Md* = 1, *n* = 34), *U* = 2033, *z* = 3.335, *p* = .001).

**Conclusions:**

This study reveals the association between nomophobia, phubbing, and depression in both cohorts, with younger individuals and women showing higher levels. It is therefore important to conduct more robust studies and develop awareness and mental health prevention initiatives.

**Disclosure of Interest:**

I. Faria: None Declared, B. Maia Grant / Research support from: UIBD/00683/2020, FCT

